# A case of extragastrointestinal stromal tumor complicated by severe hypoglycemia: a unique presentation of a rare tumor

**DOI:** 10.1186/s12885-016-2968-8

**Published:** 2016-12-01

**Authors:** Zeb Saeed, Solaema Taleb, Carmella Evans-Molina

**Affiliations:** 1 Departments of Medicine, Indiana University School of Medicine, Indianapolis, USA; 2 Celllular and Integrative Physiology, Indiana University School of Medicine, Indianapolis, USA; 3 Biochemistry and Molecular Biology, Indiana University School of Medicine, Indianapolis, USA; 4 Herman B Wells Center for Pediatric Research, Indiana University School of Medicine, 635 Barnhill Drive MS 2031A, Indianapolis, IN 46202 USA; 5 Roudebush VA Medical Center, Indianapolis, IN 46202 USA

**Keywords:** Tumor induced hypoglycemia, Extragastrointestinal stromal tumor (GIST), Non-islet cell tumor hypoglycemia

## Abstract

**Background:**

Non-Islet Cell Tumor Hypoglycemia (NICTH) is a rare paraneoplastic cause of hypoglycemia arising from excess tumor production of insulin-like growth factor. The objective of this report is to describe an unusual case of Extragastrointestinal Stromal Tumor (EGIST) associated NICTH.

**Case presentation:**

A 64 year-old African female was brought to the emergency room with a 1-month history of recurrent syncope, weight loss, and abdominal bloating. Serum blood glucose was discovered to 39 mg/dL, when insulin, proinsulin, and C-peptide were suppressed. Computed tomography scan revealed a diffuse extraintestinal metastatic disease process, and a biopsy confirmed the diagnosis of an Extragastrointestinal Stromal Tumor (EGIST). IGF-I and II levels were 27 ng/ml and 262 ng/ml respectively, and the ratio of IGF-II to IGF-I was calculated as 9.7:1, suggestive of IGF-II-mediated NICTH. Acutely, the patient’s hypoglycemia resolved with dextrose and glucagon infusion. Long-term euglycemia was achieved with prednisone and imatinib therapy.

**Conclusions:**

NICTH should be considered when hypoglycemia occurs in the setting of low serum insulin levels. Whereas definitive treatment of EGIST involves surgical resection, immunotherapy with tyrosine kinase inhibitors and corticosteroids have been shown to alleviate hypoglycemia in cases where surgery is delayed or not feasible.

## Background

Tumor induced hypoglycemia can be divided into two broad categories. The first involves insulin hypersecretion from pancreatic islet cell insulinomas. The second, known as Non-Islet Cell Tumor Hypoglycemia (NICTH), is from paraneoplastic production of insulin-like growth factor from a tumor, leading to unrestrained glucose uptake at peripheral tissues [1, 2]. The first description of NICTH dates back to 1929 and involved a patient with metastatic hepatocellular carcinoma. Post-mortem examination of the pancreas was normal. Furthermore, analysis of the tumor failed to reveal the presence of insulin, thus leading to the conclusion that the hypoglycemia was non-insulin mediated [3]. Since this original description, a variety of tumors have been shown to exhibit NICTH. These primarily include tumors of mesenchymal and epithelial origin with hepatocellular carcinomas being among the most frequently implicated.

Gastrointestinal Stromal Tumor (GIST) is the most common mesenchymal tumor arising within the gastrointestinal (GI) tract. These tumors express the phenotype of the Interstitial Cells of Cajal or related stem cell-like precursors and are associated with somatic mutations of the tyrosine kinase receptors c-kit (CD117) and platelet-derived growth factor-α (PDGFR-α) [4–6]. Over the last decade, a handful of case reports have described an association between GIST and NICTH [5]. In extremely rare cases, GIST can arise primarily outside the GI tract, where it is termed Extragastrointestinal Stromal Tumor (EGIST) [6, 7]. Representing less than 10% of all stromal tumors, EGISTs share the same histological features, immunophenotype, and biological behavior as GISTs. Most EGISTs originate in the lesser or greater omentum, the mesentery, or less commonly in the retroperitoneum, with very few cases reported of tumors arising from the abdominal wall itself [6, 8]. While some sources suggest that EGISTs represent peritoneal metastases of undiagnosed GISTs or GISTs that may have detached from the intestinal wall during extensive extramural growth, others consider them to be primary tumors arising from multipotent mesenchymal stems cells of the extra-intestinal tissue [6, 8, 9]. Surgery remains a mainstay for localized GIST/EGIST. However, immunotherapy with tyrosine kinase inhibitors (TKI), especially imatinib, has emerged as a promising neoadjuvant or alternative therapy.

Here, we describe the case of a patient presenting with a rare abdominopelvic EGIST tumor and recurrent episodes of severe symptomatic hypoglycemia. To the best of our knowledge, this is the first reported case linking EGIST and NICTH.

## Case Presentation

A 64 year-old African female was brought to the emergency room in January 2016 with a chief complaint of recurrent syncope. Her serum blood glucose on arrival was 39 mg/dL. A detailed review of systems was notable for non-specific abdominal bloating and distension and a 50 lb weight loss over the preceding year. Vital signs on presentation were within normal limits; physical exam revealed a firm palpable right lower quadrant mass. Her past medical history revealed a history of pelvic EGIST that had been diagnosed in March 2010 at an outside facility. A computed tomography (CT) scan at that time demonstrated a large pelvic mass involving the right ovary, the mesovarian, and the mesometrium. Pathological analysis of the tumor revealed diffuse staining for c-Kit, CD-34, and the smooth muscle marker caldesmon, while stains for pancytokeratin, S-100, Human Melanoma Black (HMB-45), Melan-A, actin, myogenin and desmin were all negative. The mitotic index was noted to be high at 28/50 high power fields, and tumor necrosis was noted. Following a debulking procedure, all intestinal specimens were found to tumor-free. Hence, the diagnosis of a primary EGIST was made. Post-operatively, she was started on immunotherapy with the TKI, imatinib. Since that time, the patient missed most of her follow-up appointments. During the current admission, she reported discontinuation of imatinib several months ago. She further noted that hypoglycemia had indeed been the initial manifestation of her EGIST in 2010, but this symptom had resolved post-operatively.

**Fig. 1:**
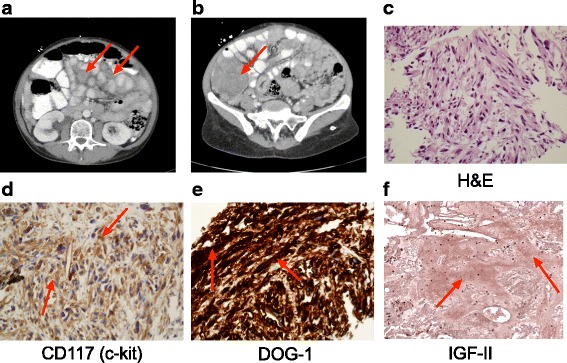
Imaging and Pathologic Analysis of the Tumor. **a**-**b** Abdominal CT scan revealed diffuse intraabdominal metastatic disease. **c** Hematoxylin and eosin (H&E) staining of the tumor revealed a spindle cell morphology (40X). Tumor immunostaining revealed the presence of **d** CD117 (40X); **e** DOG-1 (40X); and **f** IGF-II (20X)

During the current admission, initial inpatient evaluation revealed a suppressed insulin (<1uIU/mL), pro-insulin (<1.4pmol/L), C-peptide (0.4 ng/mL) and β-hydroxy butyrate (0.4 mg/dL), when the serum blood glucose was 26 mg/dL. A urine sulfonylurea screen was negative, and adrenal insufficiency was ruled out following documentation of a normal cortisol response to cosyntropin stimulation. A repeat CT scan revealed evidence of a diffuse metastatic disease with innumerable soft tissue peritoneal nodules of various sizes scattered throughout the abdomen (Fig. 1a). The largest mass was seen in the right lower quadrant and was >7 cm in the largest dimension (Fig. 1b). Given biochemical and imaging findings, consideration for EGIST-associated NICTH was made. IGF-I and II levels were assayed and found to be 27 ng/ml (reference range: 75–263 ng/mL) and 262 ng/ml (reference range: 47–350 ng/mL), respectively. The ratio of IGF-II to IGF-I was calculated as 9.7:1.

Fine needle aspiration and core biopsy of the right lower quadrant mass were performed. H&E staining revealed the typical spindle shaped morphology (Fig. 1c). Immunostaining for CD117 (Fig. 1d) and Discovered on GIST-1 (DOG-1) (Fig. 1e) were diffusely positive. Muscle specific actin (HHF-35), a mesenchymal marker present in 47% of GIST biopsies, was positive, while immunostaining for carcinoma markers were negative (not shown). Hence, a diagnosis of recurrent metastatic EGIST was made. Tumor sections were next stained with an antibody that recognized both big IGF-II and mature IGF-II (Abcam, Cambridge MA). Consistent with the patient’s presentation with severe hypoglycemia, the tumor stained positive for big IGF-II/IGF-II (Fig. 1f).

## Discussion

The true incidence of NICTH is unclear, but has been estimated to be extremely rare at approximately one per one million person years [10]. Hypoglycemia is thought to arise from excessive tumor production of IGF-II or its precursor high molecular weight “big” IGF-II. Hence the term “IGFII-oma” is often used to describe these tumors [1]. Both IGF-I and IGF-II are structurally related to insulin and act at peripheral insulin receptors to mimic insulin action, leading to increased glucose uptake, suppression of lipolysis, and inhibition of glycogenolysis, gluconeogenesis, and ketogenesis in the liver [11].

Under normal conditions, the liver produces IGF-II, in a growth hormone-independent manner, and IGF-II forms a biologically inactive ternary complex with IGF binding proteins (IGFBP), the most common of which are IGFBP3 and acid labile subunit (ALS) [12, 13]. Tumor-produced IGF-II has an equal affinity for IGFBPs compared to IGFI and II. However, instead of forming an inactive ternary complex, IGF-II forms smaller binary complexes with IGFBP. In NICTH, there is an increase in the quantity of unbound and active IGF-II as well as increased formation of these active binary complexes, which exhibit increased vasculature permeability and significantly increased bioavailability. As a side mechanism, increased IGF-II in turn further suppresses hepatic IGFBP production, thus propagating further insulin like activity and creating a vicious cycle of severe and symptomatic hypoglycemia [12].

NICTH is typically a diagnosis of exclusion, but should be suspected when hypoglycemia without hyperinsulinemia is present. Biochemically, insulin, proinsulin, C-peptide, growth hormone and β-hydroxybutyrate levels are low at the time of hypoglycemia, as was observed in this patient. In contrast, IGF-II or IGF-II precursor levels are often elevated. Typically an IGF-II:IGF-I ratio of 10:1 is considered pathognomonic, while a normal ratio is around 3:1 [1, 12]. In our patient, the levels were 9.7:1, which were quite high, though perhaps not diagnostic.

The range of tumors associated with NICTH is broad. In recent years, a handful of case reports and case series have described GIST-associated NICTH [4, 9, 11]. GIST tumors arise from the gut wall. In rare instances, they arise outside the GI tract in the omentum, mesentery, and retroperitoneum and are referred to as EGISTs. EGISTs share the same histological and immunotypic features and biological behavior as GISTs [4, 14]. The diagnosis of GIST/EGIST tumors relies on CD117 (c-KIT) positivity on immunohistochemical staining, which remains a highly sensitive and specific marker.

Discovered on GIST-1 (DOG1) protein is another recently discovered tumor marker that has particularly utility in identifying tumors harboring mutations in platelet-derived growth factor-α (PDGFR-α) [15]. Whereas staining for both CD117 and DOG1 were observed in this patient’s tumor, formal mutation analysis was not performed. Surgery remains the mainstay for localized GIST/EGIST. However, immunotherapy with tyrosine kinase inhibitors (TKI), especially imatinib, has emerged as a promising neoadjuvant or alternative therapy. TKIs are especially useful in surgically unresectable or malignant tumors, and the advent of TKIs has increased median survival in such cases by nearly 50% [16, 17].

Besides resection and therapy aimed at shrinking the underlying tumor, management of NICTH is typically independent of its location. A variety of approaches have been described in literature, including steroids, octreotide and human recombinant growth hormone (hGH) [18, 19]. Notably, here we describe the use of continuous glucagon infusion as an effective treatment in the acute setting, especially when intravenous dextrose is ineffective alone. Among long-term interventions, glucocorticoids have demonstrated the most efficacy in terms of reversing the biochemical abnormalities associated with tumor production of IGF-II [18, 20]. The proposed mechanism of glucocorticoids is thought to be suppression of IGF-II production by the tumor and/or its increased sequestration. Indeed published studies have demonstrated a significant fall in circulating big IGF-II, accompanied by an increase in serum ALS in response to prolonged glucocorticoid use. Interestingly, these changes were found to be reversible with glucocorticoid withdrawal. Furthermore, the required dose of steroid appears to be somewhat individualized and based on both tumor debulking strategies and the co-administration of other agents [18, 20]. Table 1 summarizes available published studies in which steroids were used either solely or in conjunction with other therapies in cases of inoperable NICTH (Table 1). In aggregate, the initial dose of glucocorticoid was at least 30 mg or its steroid equivalent dose, with attempts made to taper therapy based on the maintenance of euglycemia [19–21]. The minimum effective daily dose in cases employing only steroids was 20 mg once daily or 5 mg three times a day, with further dose reductions resulting in relapse of hypoglycemia [18, 20]. The ideal duration of therapy remains unclear from the literature, with most studies having variable follow-up.

**Table 1:** Use of steroids in the treatment of inoperable NICTH

Study	Number of patients	Glucocorticoid used (and dosages)^a^	Duration of therapy	Goal of the study
Teale, et al. 1998 [18]	4	One patient was treated with dexamethasone 4 mg three times a day. Three patients were treated with prednisolone 30 mg once daily.	6 weeks–7 months	To compare the effectiveness of hGH and glucocorticoid therapy in NICTH by analyzing the molecular distribution of different forms of IGF-II and IGFBP-3.
Teale, et al. 2004 [20]	6	Five patients were treated with prednisolone. Different regimens included: 20 mg once daily; 30 mg once daily; 5 mg three times a day) One patient was treated with dexamethasone 4 mg once daily.	6–13 months	To compare the outcome of different treatment options in NICTH
Bourcigaux, et al. 2005 [9]	1	Three different phases of study: phase 1 with prednisone only, phase 2 with hGH only, phase 3 with both. The lowest effective dose when steroids were used alone was prednisone 30 mg.	Days (experimental study)	To assess if combination therapy with low dose steroid and hGH is effective in inoperable patients with NICTH.
Perros, et al. 1996 [19]	1	Prednisone 30 mg once daily given with a combination of hGH and bendrofluazide; steroids were lowered later to 15 mg once daily (only in combination),	9 months	Case Report

^a^Only the dose which achieved and maintained euglycemia is included

Our patient continued to have severe hypoglycemia as an inpatient. This was acutely treated with continuous infusions of both glucagon and dextrose. Once the diagnosis of recurrent NICTH was established, she was successfully transitioned to prednisone 40 mg once daily, and euglycemia was achieved. Since surgical resection was not a viable option given the extent of her disease, imatinib was restarted after discussion with oncology with the aim of decreasing tumor burden. Over the next few months, she was successfully weaned off all steroids and repeat imaging demonstrated some shrinkage of the tumor with TKI therapy.

## Conclusion

In summary, NICTH is a rare paraneoplastic phenomenon that has been described in association with a variety of neoplasms. The pathophysiology of NICTH arises from excessive tumor production of both IGF-II and its precursor “big” IGF-II, resulting in an altered IGF-I to IGF-II ratio and insulin-independent stimulation of the insulin receptor. While cases of GIST associated NICTH have been reported, to our knowledge, our patient remains distinctive in having an extremely rare extragastrointestinal GIST tumor with an even more rare presentation of non-islet cell tumor hypoglycemia. While definitive treatment involves tumor resection and/or adjuvant treatment with TKIs, corticosteroids have been shown to successfully alleviate hypoglycemia. The overall consensus from a limited number of published reports suggests initiating steroids at a dose of prednisone 30 mg or higher and then tapering over weeks to months to the smallest dosage required to maintain euglycemia [18].

## Abbreviations

CD117: Tyrosine kinase receptors c-kit
CD34: Hematopoetic progenitor cell antigen CD34
CT: Computed tomography
DOG1: Discovered on GIST-1
EGIST: Extragastrointestinal stromal tumor
GIST: Gastrointestinal stromal tumor
hGH: Human recombinant growth hormone
HHF-35: Monoclonal muscle specific actin antibody
HMB-45: Human melanoma black
IGFBP: IGF binding proteins
IGF-I: Insulin like growth factor I
IGF-II: Insulin like growth factor II
Melan-A: Melan-A/MART-1
NICTH: Non-islet cell tumor hypoglycemia
PDGFR-α: Platelet-derived growth factor-α
S-100: S-100 protein
TKI: Tyrosine kinase inhibitor
